# Clinical and Genetic Characterization of a Russian Family with Bardet–Biedl Syndrome Carrying a Previously Undescribed Missense Variant and a Recurrent Pathogenic Frameshift Variant in *BBS7* Gene

**DOI:** 10.3390/genes17060614

**Published:** 2026-05-29

**Authors:** Alexandra F. Nikolaeva, Timur V. Boyko, Vitaly V. Kadyshev, Elena A. Shestopalova, Svetlana V. Kuznetsova, Elizabeth G. Panchenko, Anatoly N. Tiulpakov, Oxana P. Ryzhkova, Tamara O. Aripova, Tatyana R. Hegay, Sergey I. Kutsev, Vladimir O. Sigin

**Affiliations:** 1 Research Centre for Medical Genetics, Moskvorechye st., 1, Moscow 115522, Russia; boyko.timur@inbox.ru (T.V.B.); vvh.kad@gmail.com (V.V.K.); svetakuz123321@gmail.com (S.V.K.); pangen1994@gmail.com (E.G.P.); sigin.vladimir@gmail.com (V.O.S.); 2 Faculty of Medical Biology, Department of Medical Biochemistry, Pirogov Russian National Research Medical University, Ostrovityanova st., 1, Moscow 117997, Russia; 3 Moscow Clinical Scientific Center named after A. S. Loginov, 1 Novogireevskaya st., bldg. 1, Moscow 111123, Russia; 4 Morozovskaya Children’s Clinical Hospital of the Moscow City Healthcare Department, 4th Dobryninskiy Per., 1/9, Moscow 119049, Russia; 5 Department of General and Medical Genetics, Pirogov Russian National Research Medical University, Ostrovityanova st., 1, Moscow 117997, Russia; 6 Russian Children’s Clinical Hospital, Leninsky Pr., 117, Moscow 119571, Russia; 7 Institute of Immunology and Human Genomics, Academy of Sciences of the Republic of Uzbekistan, 74 Yahyo Gulyamov Street, Tashkent 100060, Uzbekistan; 8 Tashkent Research Centre for Medical Genetics, 74 Yahyo Gulyamov Street, Tashkent 100060, Uzbekistan

**Keywords:** BBS7, Bardet–Biedl syndrome, obesity

## Abstract

**Background/Objectives:** Bardet–Biedl syndrome (BBS) is a rare autosomal recessive ciliopathy caused by variants in genes encoding components of the BBSome complex. Interpretation of rare variants in the *BBS7* gene remains challenging, particularly for variants of uncertain significance. This study aimed to provide clinical and molecular genetic characterization of two siblings with a BBS phenotype harboring compound heterozygous variants in *BBS7*, including a previously undescribed missense variant. **Methods:** Two siblings (aged 11 and 8 years) presenting with obesity, postaxial polydactyly, retinal dystrophy, and cystic renal dysplasia were clinically evaluated. Whole-exome sequencing was performed in the proband, followed by segregation analysis within the family. Variant classification was conducted according to ACMG/AMP guidelines, integrating allele frequency data, in silico predictions, segregation evidence, and structural modeling. **Results:** Two heterozygous variants in the *BBS7* gene (NM_176824.3) were identified in trans: a previously reported pathogenic frameshift variant, c.1967_1968delinsC, p.(Leu656ProfsTer18), and a missense variant, c.454T>C, p.(Cys152Arg), which, to the best of our knowledge, has not been previously reported in patients with Bardet–Biedl syndrome. The variant was extremely rare in population databases. Structural analysis suggested steric and electrostatic disruptions, including loss of a disulfide bond. Based on ACMG/AMP criteria, the variant was classified as likely pathogenic. **Conclusions:** This case supports the likely pathogenic classification of the c.454T>C, p.(Cys152Arg) variant. The findings highlight the importance of integrating clinical, genetic, and structural data for the interpretation of rare variants in BBS-associated genes.

## 1. Introduction

Bardet–Biedl syndrome (BBS) is an autosomal recessive ciliopathy resulting from genetic defects in proteins essential for the structural and functional integrity of primary cilia [1]. The clinical phenotype of BBS is characterized by marked variability and includes early-onset retinal dystrophy, early-onset obesity, postaxial polydactyly, cognitive impairment, hypogonadism, and renal anomalies [2]. The prevalence of BBS shows marked geographic variability, ranging from approximately 1:100,000 to 1:160,000 live births, while in certain isolated communities it may increase to 1:13,500–1:18,000 [3]. The pathogenic mechanism of BBS is driven by primary ciliary dysfunction resulting from defects in genes encoding proteins involved in intraflagellar transport (IFT), a form of intracellular transport responsible for the biogenesis and maintenance of primary cilia [4,5]. The molecular organization of IFT is mediated by specialized protein complexes within cilia, in particular the BBSome complex and the BBS chaperone complex [6].

To date, at least 28 genes have been identified as causally associated with BBS or involved in its pathogenesis [3,5]. The mutational burden is highest in the *BBS1*, *BBS2*, *BBS4*, *BBS5*, *BBS7*, *BBS9*, *BBS10*, and *BBS12* genes, which together account for approximately 70–80% of all molecularly confirmed cases. Among Russian patients with BBS, a distinct feature has been observed: pathogenic and likely pathogenic variants in the *BBS7* gene account for up to 24% of cases, substantially exceeding the corresponding global estimate (approximately 1.5–2%) [7,8,9]. A recurrent variant, NM_176824.3(*BBS7*):c.1967_1968delinsC, p.(Leu656ProfsTer18), has been identified in this gene in multiple unrelated patients, suggesting a founder effect in the Russian Federation [10].

The *BBS7* gene is mapped to locus 4q27; its product, the BBS7 protein, is a key component of the BBSome complex required for both anterograde and retrograde transport of molecular cargo [11]. The presence of a mutant BBS7 subunit within the BBSome leads to dysfunction of the entire complex. In *Bbs7* knockout mouse models, the structure of primary cilia and the IFT complex is preserved; however, similarly to *Bbs2*^−/−^ and *Bbs4*^−/−^ models, abnormal accumulation of the dopamine receptor D1 is observed in the ciliary membrane, indicating a role for BBS7 in the specific localization of membrane proteins to primary cilia [12]. Moreover, the absence of functional BBS7 leads to the accumulation of Smoothened (Smo) in primary cilia, resulting in aberrant activation of the Hedgehog (Hh) signaling pathway and contributing to the associated phenotypic manifestations, including dental anomalies [13,14].

According to the HGMD Professional database (version 2025.1), the variant spectrum of the *BBS7* gene is dominated by missense variants and frameshift mutations; however, the application of modern approaches, particularly whole-genome sequencing, may reveal an additional contribution of deep intronic and structural variants. In addition to the classical features of BBS, specific phenotypic traits have been described for *BBS7* mutations, such as bilateral accessory auricles. Furthermore, more severe renal involvement has been reported in association with mutations in the *BBS7* gene compared with other genetic forms of BBS [15,16].

Differential diagnosis of BBS includes a range of hereditary disorders characterized by the combination of obesity and retinal dystrophy, including Alström and Cohen syndromes, as well as isolated forms of retinal degeneration and postaxial polydactyly [17]. Molecular genetic verification of the diagnosis is critically important for genetic counseling, prognostic assessment, and identification of asymptomatic carriers of pathogenic variants. At the same time, the interpretation of previously undescribed or rare variants in BBS-associated genes (particularly *BBS7*) remains a major diagnostic challenge due to pronounced locus heterogeneity and the lack of functional data for a substantial proportion of missense variants. As a result, a considerable fraction of identified nucleotide variants is classified as variants of uncertain significance (VUS), complicating definitive clinical diagnosis and necessitating additional studies and segregation analysis [18]. According to current recommendations of the American College of Medical Genetics and Genomics (ACMG), an integrative approach combining allele frequency data, in silico predictive analyses, segregation data, and functional studies enables more accurate classification of variant pathogenicity [19].

In this report, we present a familial case involving affected siblings referred for genetic counseling due to progressive visual impairment, early-onset obesity, and postaxial polydactyly of both upper and lower limbs. This case contributes to expanding the mutational spectrum of the *BBS7* gene and provides additional evidence supporting the reclassification of the c.454T>C p.(Cys152Arg) variant, which is reported here for the first time. Application of ACMG variant interpretation criteria—considering its occurrence in compound heterozygosity with a known pathogenic variant, localization within an evolutionarily conserved protein domain, and consistent results from in silico prediction tools (SIFT, PolyPhen-2, and MutationTaster)—supports upgrading its pathogenicity to the likely pathogenic category. This report highlights the importance of detailed clinical-genetic correlation and the accumulation of data on rare variants in BBS-associated genes as essential for improving diagnostic accuracy and optimizing genetic counseling.

## 2. Case Presentation

An 11-year-old and 8-year-old, siblings, were referred to the Research Centre for Medical Genetics under a program for the diagnosis of early-onset severe obesity. Both were born via spontaneous vaginal delivery, with birth weights of 3.646 kg and 3.560 kg, respectively. The 11-year-old patient had a history of a congenital heart defect (moderate stenosis of the peripheral branches of the pulmonary artery) diagnosed shortly after birth. At the age of 1.5 years, he developed esotropia. In addition, he presented with postaxial polydactyly of the fifth digits of both hands and feet, pes planovalgus deformity of the right foot, congenital left-sided clubfoot, cystic renal dysplasia, grade II cerebral ischemia, and hypogonadism. The mother also reported rapid weight gain, delayed speech development, and poor academic performance. The family history was notable for the 8-year-old sibling, who had a clinical presentation consistent with BBS and similar to that of the proband.

**Figure 1:**
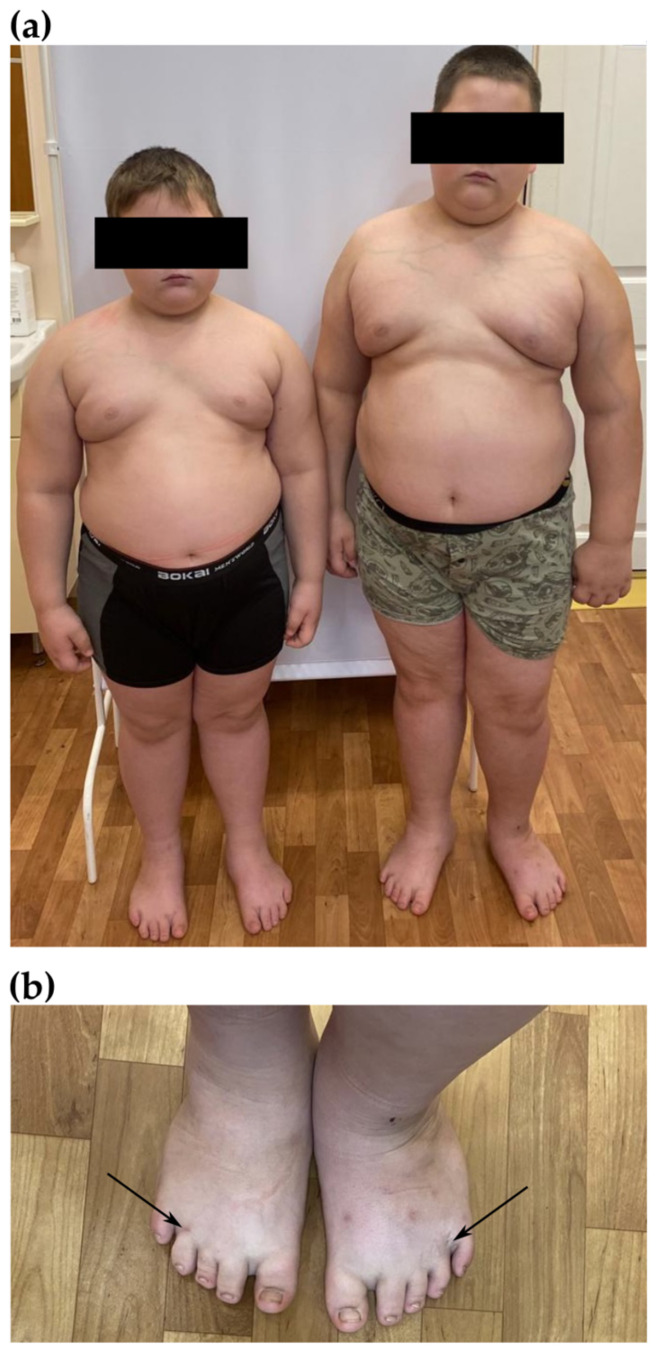
(**a**) Photographs of the siblings aged 11 (right) and 8 (left), demonstrating obesity. (**b**) Postoperative scars following correction of bilateral postaxial polydactyly of the lower limbs (indicated by arrows).

Clinical examination by a geneticist revealed characteristic phenotypic features, including obesity (BMI SDS +3.5 and +4.0, respectively), with the older sibling measuring 144 cm, 74 kg (BMI 36 kg/m^2^) and the younger sibling 136 cm, 61 kg (BMI 33 kg/m^2^), as well as a high forehead and a broad nasal tip. Both siblings had previously undergone surgical correction of postaxial polydactyly with removal of supernumerary digits of the hands and feet (Figure 1).

Laboratory investigations, including a complete blood count, fasting glucose measurement, and serum inorganic phosphorus levels, revealed no abnormalities. All parameters were within reference ranges.

In both cases, a reduction in visual function of 60–80% from age-normal values was recorded. The older sibling exhibited low-amplitude nystagmus. Color vision testing revealed a deuteranomaly-type deficiency in both affected individuals. Nyctalopia was diagnosed only in the younger sibling.

**Figure 2:**
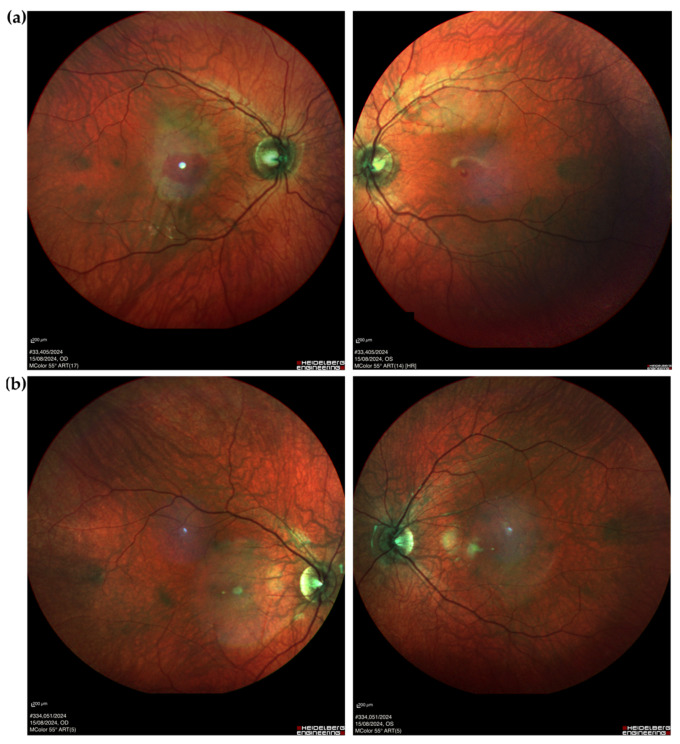
Fundus photographs of the younger affected sibling (**a**) and older affected sibling (**b**).

Biomicroscopy of the anterior segment structures revealed no pathological changes. Best-corrected visual acuity (BCVA) in the older affected sibling was 0.3 in the right eye and 0.4 in the left eye; in the younger affected sibling, BCVA was 0.2 in both eyes. Ophthalmoscopy was performed under pharmacologically induced mydriasis. The funduscopic findings were identical in the two patients: the optic disk was waxy and atrophic, of normal diameter, with well-defined margins; the capillary network and perineural ring on the disk were preserved and symmetrical. The arterial vessels were narrowed. The macular area was well differentiated, with a distinct foveal depression and a clear foveolar reflex; pigmentation was present in the fovea. In the mid- and far periphery of the fundus, choroidal vessels were visible, and pigment redistribution was observed. No bone-spicule pigmentation was detected (Figure 2).

**Figure 3:**
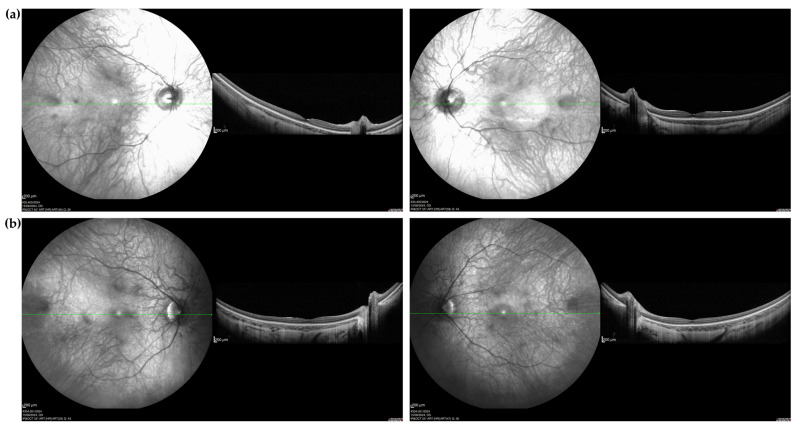
Optical coherence tomography images of the fundus structures of the younger affected sibling (**a**) and older affected sibling (**b**). The green arrow indicates the OCT scan direction on the fundus image. The corresponding B-scan represents a cross-sectional tomographic image of the retina acquired along this trajectory.

Spectral-domain optical coherence tomography (SD-OCT) of the fundus structures was performed in both siblings. In both cases, identical changes were recorded: a well-expressed foveal pit; the external limiting membrane was distinguishable; the IS/OS junction (the boundary between the inner and outer segments of the photoreceptors) was well visualized; cellular rarefaction was observed in the fovea; bilaminarity at the level of the retinal pigment epithelium (RPE)–Bruch’s membrane complex was poorly expressed (Figure 3).

Full-field electroretinography in both affected siblings revealed severely attenuated scotopic and photopic responses. Rod, maximal combined rod-cone, cone, and 30-Hz flicker responses were either non-recordable or reduced to low microvolt/submicrovolt amplitudes bilaterally. These findings indicate a severe disturbance of bioelectrical activity of the retina in the periphery and in the center, involving both rod- and cone-mediated pathways, consistent with inherited retinal dystrophy associated with Bardet–Biedl syndrome (Table 1).

Thus, based on the ophthalmological examination findings, a clinical condition consistent with cone-rod retinal degeneration was established.

The clinical diagnosis of BBS was established in both siblings by a clinical geneticist based on established diagnostic criteria [2]. Both patients exhibited four primary features: early-onset obesity, polydactyly, early retinal dystrophy, and cystic renal dysplasia detected by ultrasound. In addition, two secondary features were noted: hypogonadism and neurodevelopmental disability.

## 3. Genetic Results

To confirm the clinical diagnosis and establish the molecular genetic etiology of BBS, whole-exome sequencing was performed in the 11-year-old proband. 

Whole-exome sequencing (WES) was performed on DNA from the proband. Libraries were prepared from fragmented genomic DNA using the KAPA Hyper Prep Kit, followed by target enrichment with KAPA HyperExome probes (Roche, Basel, Switzerland). Paired-end sequencing (2 × 150 bp) was carried out on an Illumina NextSeq 500 platform (Illumina Inc., San Diego, CA, USA). Raw reads were aligned to the human reference genome GRCh38/hg38 using NGSData v.1.0.

In total, 22,893,631 reads were obtained, with a mean coverage of 53×; 97% of target regions were covered at ≥10×. The BBS7-containing region chr4:121,825,839–121,868,073 had an average coverage of 30×. Variant calling and annotation were performed according to HGVS nomenclature v.21.1.3. The identified variant was visualized in Integrative Genomics Viewer v.2.17.4 (IGV), and its pathogenicity was assessed following ACMG guidelines [19]. Additionally, CNV analysis was performed, and other known BBS/ciliopathy genes were systematically evaluated. Given the genetic heterogeneity of BBS, we also assessed whether any other potentially relevant variants were identified or excluded.

**Figure 4:**
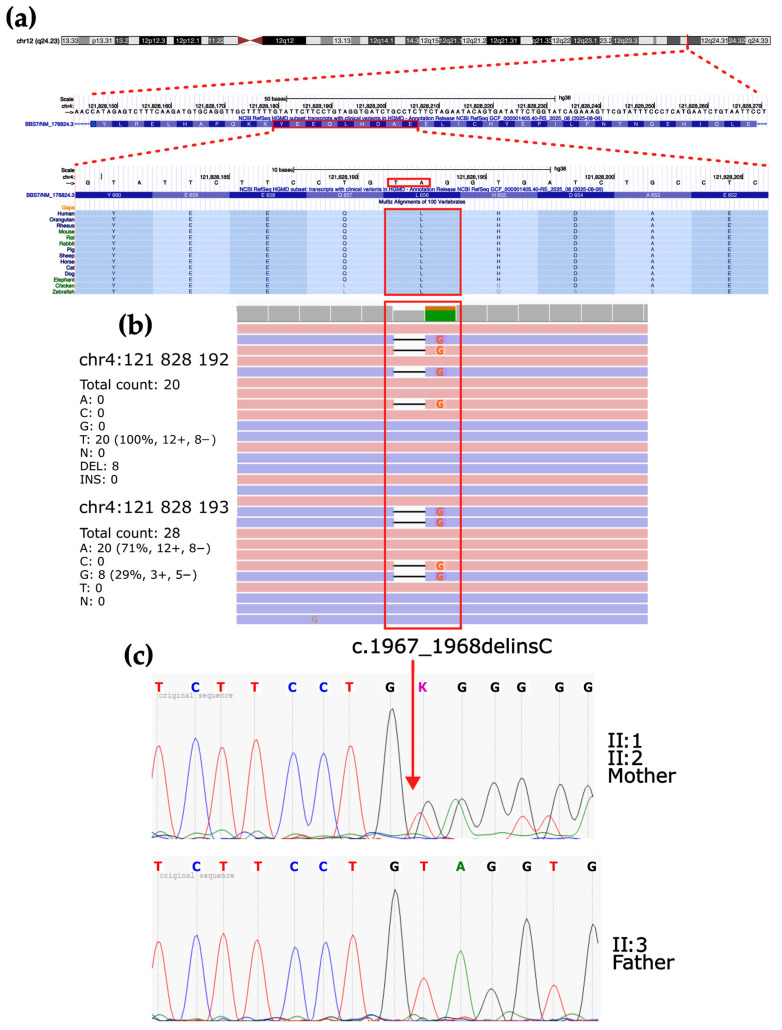
Identification and validation of a previously described *BBS7* (NM_176824.3) c.1967_1968delinsC variant. (**a**) Schematic representation of the BBS7 gene and evolutionary conservation analysis. The identified c.1967_1968delinsC, p.(Leu656ProfsTer18) frameshift variant is indicated at genomic coordinates chr4:121828192_121828193. Multiple sequence alignment across diverse species demonstrates that the leucine residue at position 656 is highly conserved (indicated by a red frame), highlighting its putative structural and functional importance. (**b**) Visualization of the heterozygous chr4:121828192_121828193delinsC variant in the *BBS7* gene using Integrative Genomics Viewer (IGV v.2.17.4). (**c**) Sanger sequencing of the proband and family members. Sanger sequencing confirmed the c.1967_1968delinsC, p.(Leu656ProfsTer18) variant in a heterozygous state in the affected siblings and in the maternal carrier. The variant is indicated by a red arrow.

**Figure 5:**
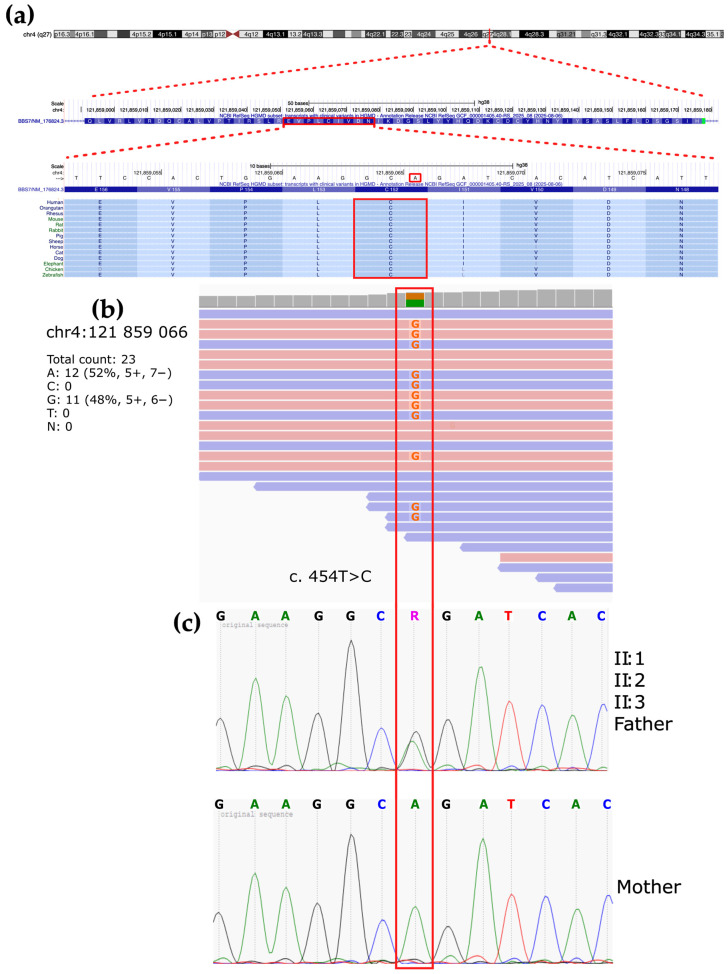
Identification and validation of a *BBS7* (NM_176824.3) c.454T>C variant. (**a**) Schematic representation of the *BBS7* gene and evolutionary conservation analysis. The identified c.454T>C, p.(Cys152Arg) missense variant is indicated at genomic coordinate chr4:121859066. Multiple sequence alignment across diverse species demonstrates that the cysteine residue at position 152 is highly conserved (indicated by a red frame), highlighting its putative structural and functional importance. (**b**) Visualization of the heterozygous chr4:121859066A>G variant in the *BBS7* gene using Integrative Genomics Viewer (IGV v.2.17.4). (**c**) Sanger sequencing of the proband and family members. Sanger sequencing confirmed the c.454T>C, p.(Cys152Arg) variant in a heterozygous state in the affected siblings and in the paternal carrier. The variant is indicated by a red frame.

Analysis identified two heterozygous variants in the *BBS7* gene (NM_176824.3): c.1967_1968delinsC, a frameshift variant resulting in a premature stop codon p.(Leu656ProfsTer18) (Figure 4), and c.454T>C, a missense variant leading to the amino acid substitution p.(Cys152Arg) (Figure 5). The c.1967_1968delinsC variant has previously been reported as pathogenic in Russian patients with BBS [20], whereas the c.454T>C variant has not been described in the literature to date.

To validate the variants identified by WES, the c.1967_1968delinsC variant was validated using PCR with the following primers, synthesized by Evrogen (Moscow, Russia): forward 5′-GATCCTTGGGAAATAGCCAGT, reverse 5′-TTTTGAATATTAAGGTGCTTCTGT; and the c.454T>C variant was validated using primers forward 5′-AGGCAGGTAAAGGTTCTCTG, reverse 5′-ACTAGGAATTAACTAGGAGAAAGTT.

PCR was performed in 25 µL reactions containing 1× PCR buffer, 2 mM MgCl_2_, 0.2 mM each dNTP, 0.5 mM each primer, 0.3 U Taq DNA polymerase (Syntol, Moscow, Russia), and 1 µL genomic DNA. Amplification was carried out on a GeneAmp PCR System 9700 Thermal Cycler (Applied Biosystems Inc., Foster City, CA, USA) under the following conditions: 95 °C for 2 min; 35 cycles of 95 °C for 30 s, 64 °C for 30 s, and 72 °C for 30 s; and a final extension at 72 °C for 5 min.

PCR products were checked on 2% agarose gels stained with ethidium bromide and visualized under UV light. Amplicons were purified using Exonuclease I/FastAP Thermosensitive Alkaline Phosphatase (Thermo Fisher Scientific, Waltham, MA, USA), then sequenced with the BigDye Terminator v3.1 kit on an Applied Biosystems 3500 DNA Analyzer according to the manufacturer’s protocol. Sequencing chromatograms were analyzed using Chromas v.2.6.6 (Technelysium, South Brisbane, QLD, Australia).

**Figure 6:**
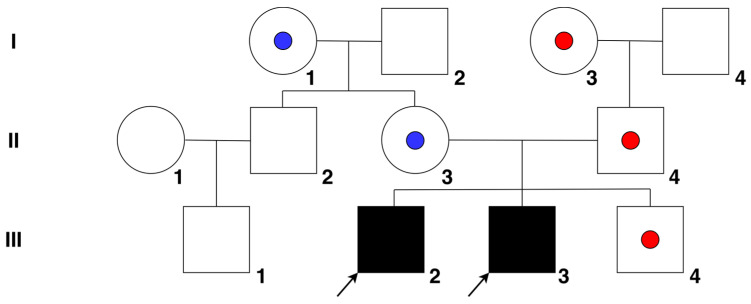
Pedigree of a family with BBS. The older and younger affected siblings are indicated by an arrow. Circles represent females, and squares represent males. Solid black symbols indicate affected individuals. Open symbols denote clinically healthy individuals who are not carriers of either variant. Family members carrying the NM_176824.3(*BBS7*):c.454T>C, p.(Cys152Arg) and NM_176824.3(*BBS7*):c.1967_1968delinsC, p.(Leu656ProfsTer18) variants are indicated in red and blue, respectively. Roman numerals (I–III) denote generations, and Arabic numerals (1–4) indicate individuals within each generation.

Segregation analysis was performed to confirm inheritance, demonstrating that the c.1967_1968delinsC variant was inherited from the mother, whereas the c.454T>C variant was inherited from the father. Accordingly, the variants are in trans. Both variants were also identified in the 8-year-old sibling with a similar clinical presentation. In addition, a 6-year-old male sibling with normal body weight and without clinical features of BBS was found to be a heterozygous carrier of the c.454T>C variant but did not carry the c.1967_1968delinsC variant (Figure 6).

## 4. Results and Discussion

Bardet–Biedl syndrome is a rare autosomal recessive ciliopathy characterized by obesity, postaxial polydactyly, retinal dystrophy, intellectual disability, and renal abnormalities [20]. Pathogenic *BBS7* variants are identified in approximately 1.5–2% of all patients with Bardet–Biedl syndrome; however, in a Russian cohort, they account for up to 24% of cases [10]. In this case report, we describe two siblings aged 11 and 8 years with Bardet–Biedl syndrome who presented with a typical clinical picture of the BBS (Table 2), harboring two heterozygous variants in trans in the *BBS7* gene. The c.1967_1968delinsC variant is not represented as a single variant in the gnomAD v.4.1.1 database; instead, it is recorded as a combination of two adjacent variants, c.1968del, p.(Gln657ArgfsTer17), and c.1967T>C, p.(Leu656Pro), each observed at a low allele frequency (0.005%). In contrast, the allele frequency in a Russian reference database (Federal Medical-Biological Agency) reaches 0.1% [21]. Moreover, it has previously been reported as pathogenic and is frequently observed in Russian patients with BBS, consistent with a possible founder effect and explaining its recurrent detection among affected individuals in Russia. [20].

The c.454T>C variant has not been described in the literature to date. The variant is present in gnomAD v4.1.1 at a very low frequency (0.0003%), with a total allele count of 5 out of 1,613,994 alleles (including 4 alleles in the European non-Finnish cohorts and 1 allele of other ancestry) and no homozygous individuals. In a Russian reference database (Federal Medical-Biological Agency), the variant was observed at a frequency of 0.004% (10 out of 241,524 alleles), also with no homozygotes [21].

**Figure 7:**
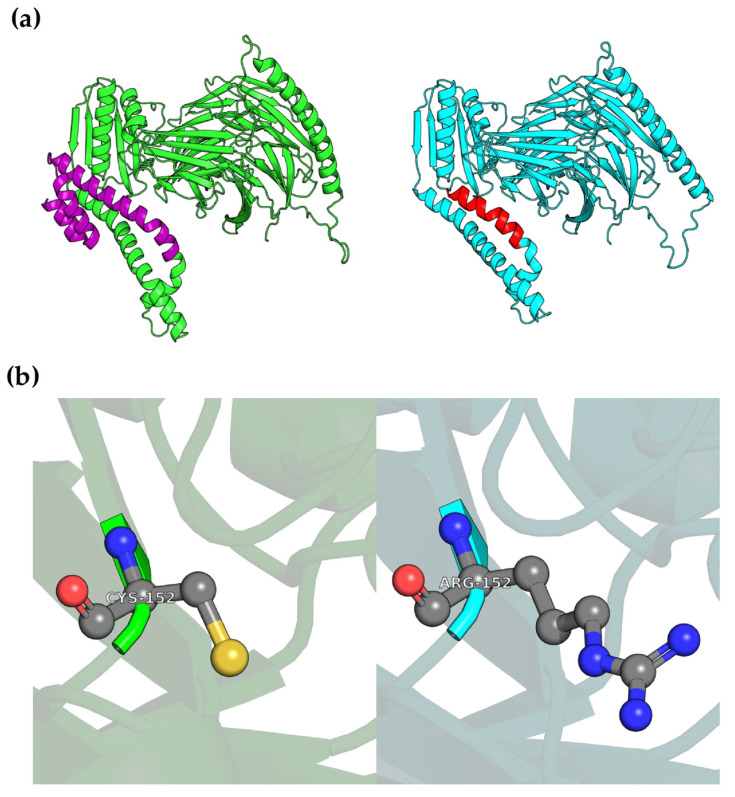
Structural modeling of the predicted effects of the missense variant in the *BBS7* gene on the tertiary structure of the protein. The PDB file of the normal (wild-type) BBS7 protein was downloaded from the UniProt database (UniProt ID: Q8IWZ6). To model the mutant protein, the coding DNA sequence (CDS) was extracted from the MANE Select Plus Clinical transcript of the *BBS7* gene (NM_176824.3). The obtained sequence was translated in silico using the ExPASy Translate service (verison) and compared with the canonical amino acid sequence of BBS7. Nucleotide substitutions (c.454T>C and c.1967_1968delinsC) were introduced into the original CDS by directly editing the sequence in a text editor, after which the translation result was verified again in silico. The amino acid sequence of the mutant protein was uploaded to ColabFold v1.6.1 (AlphaFold2 using MMseqs2), yielding an archive of predicted tertiary models with confidence scores. From this archive, the file with the highest prediction confidence was selected. The tertiary structures of both proteins (wild-type and mutant) were visualized in PyMOL v.3.1. (**a**) Left: wild-type BBS7 protein; right: BBS7 protein with the truncating variant p.(Leu656ProfsTer18). (**b**) Left: Cys152 residue in the wild-type BBS7 protein; right: Arg152 residue in the mutant BBS7 protein.

These findings confirm the previously established clinical diagnosis of Bardet–Biedl syndrome. The c.1967_1968delinsC variant in the *BBS7* gene (NM_176824.3), resulting in the p.(Leu656ProfsTer18) change, has previously been reported as pathogenic in both homozygous and compound heterozygous states in patients with Bardet–Biedl syndrome [8,10,20,26]. This variant induces a frameshift starting at codon 656 and introduces a premature stop codon (Ter) 18 residues downstream of leucine (Leu). Such truncation of the polypeptide chain is predicted to result in the loss of key protein domains, thereby impairing BBSome assembly and function (Figure 7a). The c.454T>C variant is predicted to be likely pathogenic by multiple in silico tools for missense variant interpretation (SIFT, SIFT4G, MetaLR, MetaSVM, DEOGEN2, M-CAP, PROVEAN, LRT, PolyPhen-2 HDIV, PrimateAI, and fathmm-MKL coding), which provide supportive, but not conclusive, evidence for pathogenicity. It can be assumed that the p.(Cys152Arg) substitution in the BBS7 protein leads to significant steric constraints due to the substantial difference in the side-chain volumes of cysteine and arginine: the bulkier arginine residue is likely unable to be accommodated within the protein’s tertiary structure without introducing unfavorable torsional angles and steric clashes. In addition, the substitution eliminates a cysteine residue and may affect local structural stability; however, this prediction requires experimental validation (Figure 7b).

**Figure 8:**
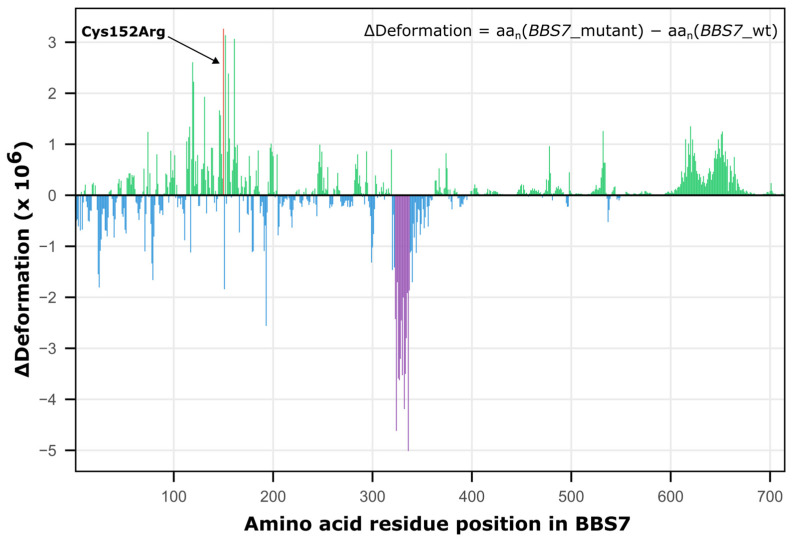
Amino acid residue-based differential structural deformation profile between the Cys152Arg mutant BBS7 and the wild-type BBS7. The X-axis displays the amino acid residue index of the BBS7 protein sequence. The Y-axis displays the ΔDeformation values (×10^−6^) between the Cys152Arg mutant BBS7 protein and the wild-type BBS7 protein. Positive values (green bars) indicate regions of increased structural deformation (higher flexibility) in the mutant protein, whereas negative values (blue bars) represent regions of structural relaxation (higher rigidity) compared to the wild-type. The site of the amino acid substitution (C152R) is marked in red, while the violet bars highlight the region (323–37) of the mutant BBS7 exhibiting the most prominent structural relaxation. Structural dynamics were analyzed using NMA based on an Elastic Network Model (ENM). Calculations were performed using the Bio3D package v.2.4-5 in R. The structural deformation profile for each residue was calculated by summing the atomic fluctuations derived from the first three non-trivial normal modes for both the wild-type and the C152R mutant BBS7 structures. aa—amino acid residue, wt—wild-type.

The Cys152Arg substitution in the BBS7 protein induces local structural perturbations that are not confined to the mutation site. As demonstrated by Normal Mode Analysis (NMA), the mutation exerts a significant long-range allosteric effect (Figure 8). The differential structural deformation profile reveals that this instability manifests as a prominent structural relaxation cluster in the 323–337 residue range, indicating a global reorganization of the protein’s dynamical network. This disruption of the native structural dynamics may impair the functional integrity of the BBS7 protein and its interaction with the BBSome complex, although these potential functional consequences require further experimental validation.

In conclusion, according to ACMG/AMP guidelines, the *BBS7* variant (NM_176824.3):c.454T>C, p.(Cys152Arg) is classified as likely pathogenic [19] (Table 3).

According to the literature, analysis of the BBS7 protein sequence does not allow definitive conclusions regarding its cellular function, as the protein shows no homology to functionally characterized proteins or known functional domains. Nevertheless, comparative analysis of BBS7 with other members of the BBS protein family suggests a possible structural relationship with BBS1 and BBS2. In particular, the BBS7 region spanning amino acids 129–196 shows similarity to the region of BBS2 between residues 147–210 and to the region of BBS1 between residues 314 and 377 [27]. The variant identified in this study is located at position 152 of the amino acid sequence. Its localization within an evolutionarily conserved region that is homologous to BBS2 and BBS1 supports its classification as likely pathogenic, as it is expected to disrupt the structure and function of BBS7. Together with segregation data and allele frequency evidence, this supports its contribution to the development of Bardet–Biedl syndrome. Notably, missense variants affecting the BBS7 region spanning amino acids 129–196 have been reported in the ClinVar database (Variation IDs: 1487264, 216828, 3346200), and additional pathogenic/likely pathogenic variants near residue 152, including loss-of-function and missense variants, are also listed (Variation IDs: 2794762, 982871, 1069619).

In previous studies using a *Danio rerio* model, BBS-associated variants in a region of the BBS1 protein homologous to BBS7 were shown to reduce both the length of primary cilia (to approximately 70% of control values) and their number (to approximately 42% of control values), and to induce phenotypic abnormalities in *Danio rerio*, including reduced eye size, shortening of the body axis, and other defects in gastrulation [28,29]. Thus, localization of the *BBS7* variant (NM_176824.3):c.454T>C, p.(Cys152Arg) within an evolutionarily conserved region homologous to domains of BBS1 and BBS2 provides additional structural and functional support for its pathogenicity.

Thus, the previously unreported missense variant p.(Cys152Arg) in compound heterozygosity with a known pathogenic frameshift variant in the *BBS7* gene provides novel information regarding the genotype–phenotype correlation. This observation expands the known mutational spectrum of *BBS7* and demonstrates for the first time that a missense variant at position 152, when combined with a frameshift allele, results in a phenotype consistent with Bardet–Biedl syndrome. These findings suggest that even moderately conserved amino acid substitutions in functionally important domains of the BBS7 protein may potentially impair its function in the absence of a normal allele.

Ultimately, the main limitations of our work are: the absence of functional verification of the novel missense variant; segregation analysis confined to a single family (three generations); technical constraints of whole-exome sequencing (incomplete coverage of non-coding regions and failure to detect large structural rearrangements); inadequate assessment of the population frequency of the identified variant; and a single case (one proband). Therefore, the present conclusions warrant validation in independent cohorts and via experimental models.

## 5. Conclusions

We present a case of two siblings (11 and 8 years of age) with Bardet–Biedl syndrome associated with compound heterozygosity for two *BBS7* variants in trans. One of these, c.1967_1968delinsC, has been previously reported as pathogenic, whereas the other, c.454T>C, represents a previously undescribed variant that we provisionally classify as likely pathogenic. Nevertheless, we caution that this classification is not definitive and will require functional validation. Because molecular confirmation may affect eligibility for approved or emerging therapies targeting specific manifestations of BBS, such as hyperphagia-associated obesity, genetic diagnosis has direct clinical relevance.

## Figures and Tables

**Table 1:** Full-field ERG findings in the older and younger affected siblings.

ERG Test	Predominant Retinal Pathway	Parameter Reported	Younger Sibling, Right Eye	Younger Sibling, Left Eye	Older Sibling, Right Eye	Older Sibling, Left Eye
Rod response	Scotopic rod-mediated response	a-marker, µV/ms	6.1/47.3	2.8/46.2	3.3/46.7	2.2/35.4
		b-marker, µV/ms	3.4/75.7	0.4/68.5	2.2/81.0	4.6/75.7
Maximal combined response	Mixed rod-cone response	a-marker, µV/ms	4.8/17.5	12.1/10.9	0.2/11.8	4.2/29.5
		b-marker, µV/ms	5.6/36.5	10.1/37.3	4.3/39.4	0.5/38.5
Cone response	Photopic cone-mediated response	a-marker, µV/ms	1.3/15.0	3.6/18.0	2.7/20.8	8.2/18.5
		b-marker, µV/ms	0.5/26.8	1.4/29.5	2.2/36.3	0.8/30.0
30-Hz flicker	Cone-mediated temporal response	Fundamental amplitude, µV	1.300	0.897	0.591	0.777

For rod, maximal combined, and cone responses, values are shown as the recorded a- and b-wave signals with the corresponding implicit time in parentheses. For 30-Hz flicker, the fundamental/base-frequency amplitude from the frequency spectrum is shown. Values originally reported in nV were converted to µV. Full-field electroretinography was performed using an EP-1000 system (Tomey Corporation, Nagoya, Aichi, Japan) with the standard ERG protocol. Recordings included dark-adapted rod responses, dark-adapted maximal combined responses, light-adapted cone responses, and 30-Hz flicker responses. Single-flash responses were recorded with 2-kHz sampling and a 1–40 Hz filter; photopic recordings were obtained against a white background of 23 cd/m^2^. For reporting, the a-marker and b-marker values and corresponding implicit times were extracted for rod, maximal combined, and cone responses, whereas the fundamental amplitude was used for 30-Hz flicker responses.

**Table 2:** BBS7-associated Bardet–Biedl syndrome: extracted clinical comparison table.

Study	Variant(s)	Population	Renal	Retinal	Obesity	Polydactyly	ND	Hypogonadism
[22]	c.1666A>C	Chinese	+	+	+	+	+	+
[20]	c.1967_1968delinsC	Russian	NR	+	+	+	+	+
[23]	c.728G>A/c.103-1G>A	Korean	+	+	+	−	+	−
[24]	c.389_390del	Chinese	+	+	+	+	+	+
[25]	c.719G>T	Pakistani	−	+	+	+	+	+
[16]	c.1002del / 4q26q27del	Chinese	+	NR	NR	+	NR	NR
Present study	c.1967_1968dlinsC/c.454T>C	Russian	+	+	+	+	+	+

The *BBS7* transcript NM_176824.3 was used. Abbreviation: “+” indicates that the feature was reported; “−” indicates that the feature was explicitly reported as absent; “NR” indicates not reported or not clearly extractable from the cited clinical description. Abbreviations: Renal, renal involvement; Retinal, retinal findings; ND, neurodevelopmental features.

**Table 3:** ACMG/AMP classification evidence for the BBS7 (NM_176824.3):c.454T>C, p.(Cys152Arg) variant, based on the 2015 ACMG/AMP guidelines and integrating clinical, genetic, and in silico data to determine its pathogenicity.

Criteria	Strength	Evidence	Limitations
PM2	Moderate	Population frequency: 0.0003% in gnomAD v4.1.1, 0.004% in a Russian cohort.	Very low frequency, but not absent.
PM3	Moderate	The variant is detected in trans with a pathogenic variant c.1967_1968delinsC; phase confirmed by parental testing.	Single family observation; possible linkage disequilibrium with another causal variant in the same gene (full gene sequencing not performed); moderate strength (not strong) according to ACMG/AMP guidelines.
PP1	Supporting	Segregation with disease in the family: two affected siblings carry both variants; parents are carriers of each variant separately. Variants segregates with 4 meiosis.	No distant relatives; possible linkage disequilibrium with true pathogenic variant; full gene sequencing (introns, UTR) not performed.
PP3	Supporting	Bioinformatic tools predict a damaging effect	In silico tools are not independent; predictions require experimental validation.
PP4	Supporting	Phenotype consistent with BBS diagnostic criteria; other BBS/ciliopathy genes excluded by WES.	Phenotype is not entirely specific to *BBS7*; clinical variability possible.

Summary classification: Combination of criteria—PM2 (Moderate) + PM3 (Moderate) + PP1 (Supporting) + PP3 (Supporting) + PP4 (Supporting). According to the ACMG/AMP 2015 guidelines. 2 Moderate and 3 Supporting lines of evidence are sufficient to classify the variant as Likely Pathogenic. Abbreviations: PM, Pathogenic Moderate; PP, Pathogenic Supporting.

## Data Availability

All original data supporting the findings of this study are included in the article. Additional information is available from the corresponding author upon reasonable request.

## Abbreviations

The following abbreviations are used in this manuscript:

ACMG/AMP	American College of Medical Genetics and Genomics/Association for Molecular Pathology
BBS	Bardet–Biedl syndrome
BMI SDS	Body Mass Index Standard Deviation Score
BCVA	Best-corrected visual acuity
CDS	Coding DNA sequence
ENM	Elastic Network Model
ERG	Electroretinography
Hh	Hedgehog signaling pathway
HGMD	Human Gene Mutation Database
IFT	Intraflagellar transport
NMA	Normal Mode Analysis
PM	Pathogenic Moderate
PP	Pathogenic Supporting
RPE	Retinal Pigment Epithelium
SD-OCT	Spectral-Domain Optical Coherence Tomography
Smo	Smoothened; transmembrane receptor component of the Hedgehog signaling pathway localized in primary cilia.
VUS	Variants of Uncertain Significance
